# External Validation of a Referral Rule for Axial Spondyloarthritis in Primary Care Patients with Chronic Low Back Pain

**DOI:** 10.1371/journal.pone.0131963

**Published:** 2015-07-22

**Authors:** Lonneke van Hoeven, Yvonne Vergouwe, P. D. M. de Buck, Jolanda J. Luime, Johanna M. W. Hazes, Angelique E. A. M. Weel

**Affiliations:** 1 Department of Rheumatology, Erasmus MC, Rotterdam, The Netherlands; 2 Department of Rheumatology, Maasstad Hospital, Rotterdam, The Netherlands; 3 Department of Public Health, Erasmus MC, Rotterdam, The Netherlands; 4 Department of Rheumatology, MC Haaglanden, Den Haag, The Netherlands; Oregon Health & Science University, UNITED STATES

## Abstract

**Objectives:**

To validate and optimize a referral rule to identify primary care patients with chronic low back pain (CLBP) suspected for axial spondyloarthritis (axSpA).

**Design:**

Cross-sectional study with data from 19 Dutch primary care practices for development and 38 for validation.

**Participants:**

Primary care patients aged 18-45 years with CLBP existing more than three months and onset of back pain started before the age of 45 years.

**Main Outcome:**

The number of axSpA patients according to the ASAS criteria.

**Methods:**

The referral rule (CaFaSpA referral rule) was developed using 364 CLBP patients from 19 primary care practices and contains four easy to use variables; inflammatory back pain, good response to nonsteriodal anti-inflammatory drugs, family history of spondyloarthritis and a back pain duration longer than five years. This referral rule is positive when at least two variables are present. Validation of the CaFaSpA rule was accomplished in 579 primary care CLBP patients from 38 practices from other areas. Performance of the referral rule was assessed by c-statistic and calibration plot. To fit the final referral rule the development and validation datasets were pooled leading to a total study population of 943 primary care participants.

**Results:**

The referral rule was validated in 579 patients (41% male, mean age 36 (sd7.0). The percentage of identified axSpA patients was 16% (n=95). External validation resulted in satisfactory calibration and reasonable discriminative ability (c-statistics 0.70 [95% CI, 0.64-0.75]). In the pooled dataset sensitivity and specificity of the referral rule were 75% and 58%.

**Conclusions:**

The CaFaSpA referral rule for axSpA consists of four easy to use predictors for primary care physicians and has a good predictive value in this validation study. The referral rule has the potential to be a screening tool for primary care by identifying CLBP patients suspected for axSpA.

## Introduction

Axial spondyloarthritis (axSpA) is relative new term in the field of rheumatology. It is a chronic inflammatory joint disease, that is potentially disabling and characterized by chronic low back pain (CLBP). [1] AxSpA is associated with increased morbidity, mortality, high health care costs and reduced work productivity. [2, 3] Quality of life and work participation can be improvement with effective treatment; non-steroidal anti-inflammatory drugs (NSAIDs) and biologicals.[4] This treatment is even more effective when it is given early in the disease course [5]. Nevertheless there is a delay of 4–9 years between the first CLBP symptoms and the final diagnosis of axSpA. [6, 7] This delay can be explained by the difficulty for primary care physicians to recognize an axSpA patient in the large amount of CLBP patients seen in primary care.

Low back pain (LBP) is one of the most common health problems and it is worldwide the largest contributor to the overall amount of years lived with disability (YLDs) causing a large burden for patients, health systems and society. [8, 9] Around 10% of LBP complaints persists for more than 12 weeks and become chronic. [10] In most countries CLBP patients are first seen by their primary care physicians. Guidelines with red and yellow flags are used to diagnose, treat and if necessary refer CLBP patients. [11]. These guidelines do not include a flag or referral recommendation specific for axSpA. The lack of a specifically axSpA flag is notable since a number of recent studies showed that up to 40% of the CLBP complaints, if patient are referred by pre-defined criteria, can be explained by axSpA. [7, 12–17] In addition to studying prevalence these studies also proposed different referral strategies. Referral strategies for axSpA aim to achieve earlier referral of patients suspected for axSpA by primary care physicians. However most of the published referral rules were not easy to use, costly, or developed in secondary care patients. This pre-selection of patients makes it hard to implement these referral strategies in primary care practice. Furthermore most published referral strategies are merely based on development studies so no external validation took place, an important step for deriving a clinical useful referral strategy. [18] In 2014 we published the CaFaSpA referral rule, a referral strategy for axSpA developed in primary care patients with CLBP and applicable for primary care physicians. [7] In this study we want to externally validate and optimize the performance of this CaFaSpA referral rule in another, independent population of young primary care CLBP patients.

## Material and Methods

### Study design and data source

We did a cross-sectional study in a large population of primary care CLBP patients from June 2011 to June 2012, the acronym of the study was the CaFaSpA (Case Finding Axial SpondyloArthritis) study. Primary-care group practices in the Rotterdam and The Hague area in the Netherlands were informed about the study and invited to participate. In total 38 GPs participated, who represented a source population of about 28.842 patients, ages 18–45 years. Potential participants with LBP were selected from the GP databases using the International Classification of Primary Care (ICPC) code L03, standing for low back pain symptom/complaint excluding radiation. [19].

From the 28.842 primary care records, 2597 (9%) patients ages 18–45 years were identified who had ever been registered by the ICPC code L03. Those 2597 patients were invited to participate by a letter on behalf of their GP. Responding participants were checked for eligibility during a telephonic interview by a research assistant. Inclusion criteria were current low back pain existing for more than 12 weeks, good understanding of the Dutch language and no contraindications for MRI (i.e. pregnancy, claustrophobia, pacemaker). Patients were excluded if there was a explainable cause for the back pain, such as a hernia nuclei pulposi or a trauma.

### Ethics statement

Written informed consent was obtained from all participants at the research center before any assessment was performed. Ethics approval from the St. Elisabeth Hospital in Tilburg, the Netherlands was obtained (NL3571806011).

### Clinical evaluation

All participants were asked to complete the ASAS [1] questionnaire on inflammatory back pain (IBP), before any clinical and/or radiological evaluation was done. This questionnaire comprised of five questions related to back pain. A positive ASAS questionnaire was achieved when four out of five questions were answered positively. The outcome the ASAS questionnaires was reported in a binary value; positive or negative. Furthermore participants completed the BASDAI[20] and ASDAS [21] questionnaire, both measure the disease activity of axSpA, a higher score indicates a higher disease activity. Also the Roland Morris disability questionnaire (RMDQ) was completed. [22] The RMDQ is a measure of disability caused by the LBP. Higher numbers on a 24-point scale reflect greater levels of disability.

Within a rheumatology setting an experienced research nurse obtained a clinical history including axSpA features, namely IBP, arthritis, psoriasis, enthesitis, dactylitis, uveitis, Crohn’s disease/colitis, good reaction to non-steroidal anti-inflammatory drugs (NSAIDs) and a positive family history of SpA.

The ‘red flags’ used by primary care physicians, standing for typical signs or symptoms that are frequently associated with specific LBP were also checked.[23] A description of the red flags is available in S1 Table. All assessments followed the definitions described in the ASAS handbook.[1] Statistical comparisons between clinical features of axSpA patients and CLBP patients were made by the Student t test or X^2^ test, when appropriate.

Blood was drawn from all patients, irrespective of the research nurse’s opinion of clinical diagnosis of axSpA or IBP, for the Erythrocyte Sedimentation Rate (normal range 0–15 mm Hg/min), C-reactive protein (normal range 1–10 mg/l) and HLA-B27 typing.

### Image evaluation

All patients underwent image evaluation by X-ray and MRI, again irrespective of the research nurse’s opinion of clinical diagnosis of axSpA or IBP. Sacroiliac joints (SIJ) were scored according to the modified New York criteria (from 0 normal, to 4 complete fusion), using conventional pelvic radiographs in the anterior-posterior view.[24] A score of 0, 1 or 2 unilateral was considered normal, while bilateral grade 2 or unilateral grade 3 or 4 was classified as positive. A definitive diagnosis of sacroiliitis on MRI was made according to the ASAS criteria: presence of a minimum amount of bone marrow edema (one lesion in at least two adjacent slices or more than one lesion in at least one slice).[25] Images were read by one out of two trained radiologists, blinded for patient identity, clinical and laboratory data. If one of the radiologists doubted the score, the two observers discussed the scan and came to consensus.

### Clinical outcome definition

Patients were classified as axSpA according to the ASAS criteria for axial spondyloarthritis. [25] Definite axSpA can be accomplished by the imaging arm; sacroiliitis on imaging (MRI or X-ray) plus ≥1 SpA feature, or by the clinical arm; no sacroiliitis on imaging but a positive HLA-B27 plus ≥2 SpA features. The SpA features are ASAS IBP, arthritis, (heel) enthesitis, uveitis, dactylitis, psoriasis, Crohn’s disease/colitis, good response to NSAIDs, family history for SpA, HLA-B27 positive and elevated C-reactive protein. A distinction between Ankylosing Spondylitis (AS) and non-radiographic axSpA (nr-axSpA) was made. The difference between AS and nr-axSpA is the presence of sacroiliits on plain radiographic of the sacroiliac joints (SI-joints). [1] AS comes with abnormalities on the X-ray consistent with sacroiliits, while nr-axSpA patients do not fulfill the imaging part of the modified NY criteria for AS.

### Validated predictors

The CaFaSpA referral rule was previously developed with logistic regression analysis and internally validated with bootstrapping and corrected for over fitting by a shrinkage factor. [7] The original regression coefficients and odds ratios are given in S1 Table. The rule contained four dichotomous variables, the ASAS IBP questionnaire (positive vs negative), family history for SpA (positive vs negative), good response to NSAIDs (positive vs negative), LBP duration (≤ 5years vs >5 years). The ASAS IBP questionnaire is positive if at least four out of five questions are answered with yes, a positive family history means a first or second degree family member with axSpA, psoriasis, Crohn’s disease/colitis or uveitis. A good response to NSAIDs implies a clear improvement or disappearance of the low back pain, within 48 hours after the start of NSAIDs treatment.

### External validation

For external validation of the referral rule, performance was assessed using discrimination and calibration measures.[26, 27] The ability to discriminate axSpA patients from CLBP patients was quantified by the c statistic, a measure for concordance. In binary outcomes, as in our model, the c statistic is identical to the area under the receiver operating characteristics (ROC) curve. Reasonable values for the area under the ROC curve range from 0.5 (no discrimination) to 1.0 (perfect discrimination). [28] Calibration is the agreement between the predicted probabilities and the observed frequencies and was assessed by estimating the calibration slope and intercept. The calibration slope is ideally 1 and reflects whether the effects of the predictors are on average correct. The calibration intercept indicates whether predictions are in general correct and is ideally 0. This intercept is assessed by fitting a logistic regression model with the linear predictor as an offset variable (setting the regression coefficients to 1). The analyses were performed using Stata version 13.0 software (Stata Corporation TX, USA) and R (version 2.15.2; The R Foundation for Statistical Computing).

### Model updating

For the model updating, we decided to combine this validation dataset and the development dataset (CaFaSpA 1 study). [7] In 2014 the development study has been published which consisted of 364 CLBP patients from 19 primary care practices who had been included from January to July 2010 from the greater Rotterdam area in the Netherlands. By combining the datasets the model is based on more patients leading to more stable predictor effects.[29] First a logistic regression analysis was performed in the combined dataset. Subsequently we tested if adding new variables to the model leaded to significant improvement of the model Chi-square, a measure for overall performance of the model.

To present the model as a referral rule, a simple scoring system was made. We rounded the regression coefficients from the logistic regression analysis of the combined model. We estimated the sensitivity and specificity for several cut points. The positive predictive value (PPV) of the chosen cut point was calculated.

## Results

**Fig 1:**
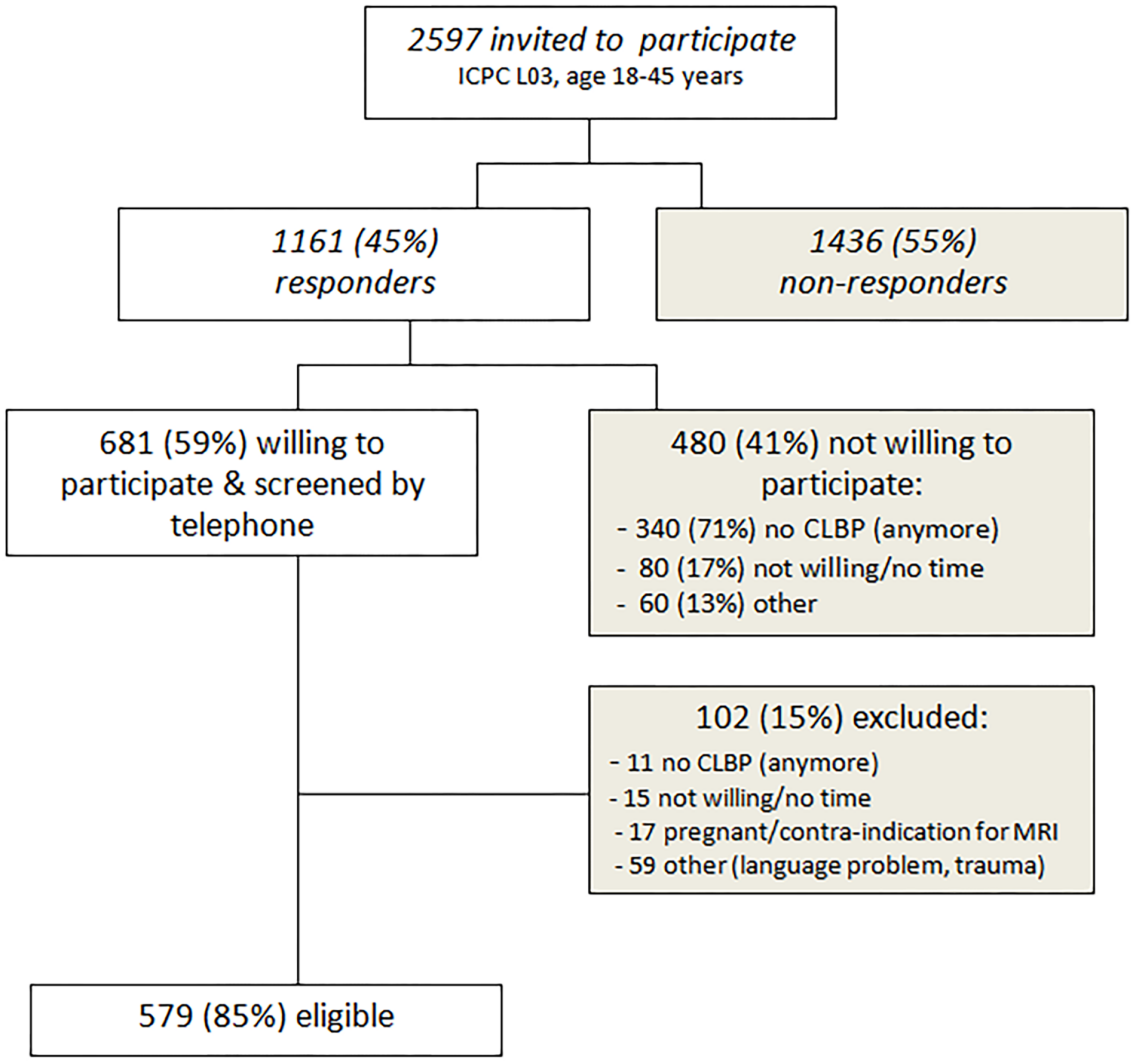
Recruitment flowchart CaFaSpA 2 study.

Out of the 2597 invited patients with low back pain, 1161 patients (44.7%) responded (Fig 1). Of these 1161 responders, 480 expressed no interest in participating and 102 did not fulfilled the inclusion criteria. Informed consent was obtained from 579 participants.

### Missing values

In the following variables missing values occurred: ASAS IBP questionnaire (1.5%) and laboratory parameters (0.5%). We assumed missing data occurred at random and performed single imputation of the variables used for the external validation.[30]

### Characteristics of the study population

Table 1 shows the characteristics of the total study population, subdivided in axSpA and CLBP patients. Overall more women (59%) participated, the mean age was 35.9 years (sd 7.0) and the median duration of low back pain was 7 years (interquartile range 3–15 years). The overall prevalence of HLA-B27 was 6.2% (n = 36). The median VAS pain was 5 (IQR 3–7), the median BASDAI and ASDAS were respectively, 4.2 (IQR 2.3–5.9) and 2.3 (IQR 1.6–3.0). The median RMDQ score was 7 (IQR 3–13). The results of the red flags are available in S1 Table. No substantial differences in characteristics between the two studies were observed.

**Table 1:** Demographics, clinical characteristics and percentage of identified axial spondyloarthritis patients of the study participants* (n = 579).

	ASAS criteria axSpA (n = 95)	Chronic low back pain (n = 484)
Age, mean ± SD years	37.3 ±6.5	35.6 ±7.1
Male sex	36 (38)	202 (42)
Caucasian	88 (93)	431 (89)
*Medical history*		
LBP duration, median (IQR) years	6.0 (4–14)	7.0 (3–15)
VAS pain, median (IQR)	4 (2–6)	5 (3–7)
ASAS IBP questionnaire (positive)†	46 (48)	147/475 (31)
Good reaction to NSAIDs	62 (65)	201 (42)
Family history SpA	24 (25)	56 (12)
IBD	1 (1)	11 (2)
Uveitis	5 (5)	18 (4)
Enthesitis	3 (3)	29 (6)
Arthritis	13 (14)	63 (13)
Dactylitis	5 (5)	14 (3)
Psoriasis	3 (3)	23 (5)
*Blood*		
CRP >10 mg/l	10 (11)	24/481 (5)
HLA-B27 positive	21 (22)	15/481 (3)
*Others*		
BASDAI, median (IQR)	4.2 (2.4–5.8)	4.2 (2.2–6.0)
ASDAS, median (IQR)	2.4 (1.7–3.0)	2.3 (1.6–2.9)
RMDQ, median (IQR)	6 (3–13)	7 (3–13)
*Percentage axSpA*		
Axial SpA	95 (16.4)	
AS	24 (25)	
Non-radiological axSpA	71 (75)	

*Values are the number (percentage) IQR = interquartile rangeLBP = low back pain; VAS = visual analog scale; ASAS = Assessment of SpondyloArthritis international Society; NSAIDs = nonsteriodal anti-inflammatory drugs; IBD = Inflammatory bowel disease; CRP = C-reactive protein; SpA = spondyloarthritis; AS = Ankylosing Spondylitis

^†^ A positive ASAS questionnaire is achieved when at least 4 out of 5 questions are answered positively.

### Percentage of identified axial spondyloarthritis patients

The percentage identified axSpA patients was 16.4% (n = 95), 95% CI: 13.5%-19.7% (Table 1). Within the axSpA cases 24 out of 95 (25%) were classified as AS and 71 (75%) as nr-axSpA. Twelve out of the 71 nr-axSpA patients (16%) fulfilled the ASAS criteria by the clinical arm, with a positive HLA-B27 status and at least two other SpA features.

### Referral rule and combining datasets

Table 2 shows the discriminative ability of the original model (c-statistic 0.70, (95%CI 0.64–0.75)). The calibration slope was 0.77 indicating that the predictor effects were on average too large. The intercept of -0.49 indicates that predictions were on average too high, which is related to the lower percentage of identified axSpA cases in the current study (16.4%), compared to CaFaSpA 1 (23.6%). The c-statistic of the combined model is 0.70, with a smaller confidence interval (95% CI 0.66–0.74).

**Table 2:** Performance of the referral rule in the validation data (CaFaSpA 2).

Performance	CaFaSpA 2 (n = 579)
C-statistic (95% CI)	0.70 (0.64–0.75)
Calibration slope (95% CI)	0.77 (0.49–1.06)
Calibration intercept (95% CI)	-0.48 (-0.73- -0.25)

The predictor effects of the ASAS IBP questionnaire, family history and reaction to NSAIDs were similar or smaller in the validation data compared with CaFaSpA 1 (Table 3). The effect of LBP duration was not profound anymore as was also shown by the similar prevalence of axSpA in two different LBP duration groups (16.8% in LBP ≤5 years versus 16.2% in LBP >5 years).

**Table 3:** Results of the multivariable logistic regression analyses in the validation data (CaFaSpA 2), development data (CaFaSpA 1) and the two data sets combined; odds ratio’s (95% confidence interval).

Predictors	CaFaSpA 2 (n = 579)	CaFaSpA 1 (n = 364)	Combined data (n = 943)
ASAS IBP questionnaire positive †	1.97 (1.24–3.13)	3.55 (2.10–5.99)	2.49 (1.77–3.50)
Family history for SpA positive	2.42 (1.38–4.24)	2.66 (1.27–5.57)	2.35 (1.51–3.65)
Good reaction to NSAIDs	2.56 (1.60–4.10)	2.42 (1.43–4.09)	2.39 (1.70–3.38)
LBP >5years	**0.78 (0.49–1.25)**	**1.96 (1.11–3.47)**	1.16 (0.82–1.64)

LBP = low back pain; NSAIDs = nonsteriodal anti-inflammatory drugs; SpA = spondyloarthritis.

^†^ A positive ASAS questionnaire is achieved when at least 4 out of 5 questions are answered positively.

We studied the additive effect of age and a dichotomized variable with easy to determine SpA features (arthritis, dactylitis, psoriasis, enthesitis, uveitis and inflammatory bowel disease, 0 = no SpA features present and 1 = ≥1 SpA feature present) in the combined data. Neither variable increased the model Chi-square significantly.

**Fig 2:**
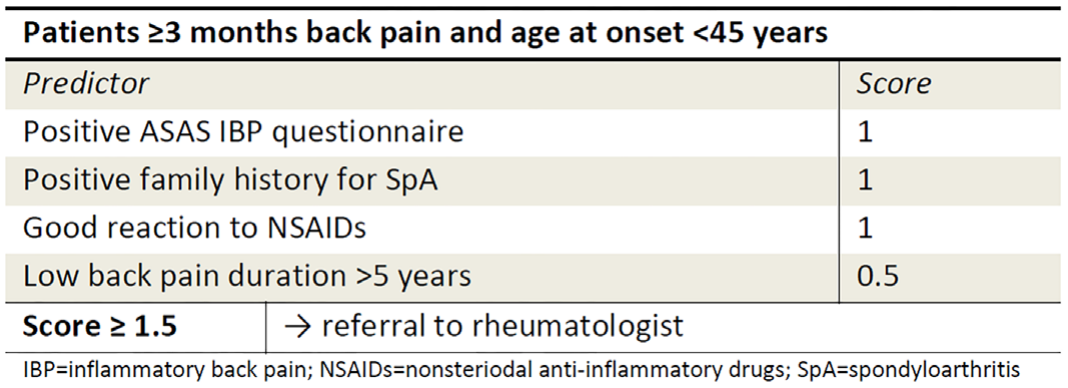
Scoring system CaFaSpA referral rule: applicable in primary care patients with chronic low back pain.

To provide a user friendly format of the prediction model, predictors with similar regression coefficients were given equal points in a simple scoring system. Fig 2 shows the combined model in this simple scoring system that can be used as a referral rule. A score of 0.5 was given to a symptom duration longer than 5 years. A positive ASAS questionnaire, a positive family history for SpA and a good reaction to NSAIDs all received one point. The cut point of 1.5 point was associated with a sensitivity of 75% and a specificity of 58% (Table 4). The yield of the referral rule expressed in the PPV is 30.2%. This means that 30.2% of the CLBP patients with a positive referral rule can be identified as axSpA.

**Table 4:** Combined model different cut points for referral rule with corresponding sensitivity and specificity.

Cut point CaFaSpA referral rule	Sensitivity (%)	Specificity (%)
≥1.0	92.3	39.1
≥1.5	74.6	57.6
≥2	40.9	82.4
≥2.5	28.7	88.3

## Discussion

Our validation study confirms the previously described high percentage of identified axSpA patients in primary care patients with CLBP. This finding emphasizes the need to introduce a simple referral strategy that can assist primary care physicians in the identification of patients with axSpA who should be referred to specialized care for diagnosis and subsequently for adequate treatment. This is the first study to externally validate a referral rule for axSpA in a primary care CLBP population.

Studying the referral rule performance in an external validation is a valuable step before implementation of the referral rule in clinical practice. Many referral rules have been developed, but only few are used in daily practice. An important reason for this discrepancy is the lack of evidence for external validity.[31] Recently several referral strategies for axSpA were published [13–17, 32], but only few have been externally validated.[13, 14] Moreover there is currently no consensus about what the most appropriate referral strategy for axSpA should be. The available referral strategies for axSpA have been developed in a pre-specified CLBP population or in already referred patients, reflected by the high prevalence of axSpA found in those studies. In contract to these studies, our study population consists of unselected primary care CLBP patients. This is the main strength of our study. Our referral rule has been validated in the population wherein the rule will be used. In our study there was no selection bias for including patients and GPs. For GPs no particular inclusion criteria were used, for patients only ICPC code L03 and age between 18 and 45 years were used, no axSpA specific inclusion criteria were required. Using ICPC code L03 comes with the disadvantage that we invited patients we aren’t currently suffering from low back pain. In ICPC code L03 no chronicity is included. This is confirmed by the finding that more than 70% of the non-participating responders didn’t had low back pain anymore (Fig 1).

The yield of our referral rule is important, the PPV of the referral rule is 30.2%. Assuming the prior probability of axSpA in a CLBP patient is 5% [33], this gives our referral rule an advantage. Our PPV is lower than the PPV of other studies [13], but our referral rule is based on clinical parameters alone. In other studies HLA-B27 testing or imaging is included in the referral strategies, which increases the PPV. However in Dutch primary care there is very limited familiarity with interpretation of SIJ imaging, and also the costs for HLA- B27 testing makes implementation of those referral strategies difficult and makes our ‘simple’ referral rule very applicable in Dutch primary care.

Three predictors from the original referral rule, the ASAS IBP questionnaire, a positive family history for SpA and a good reaction to NSAIDs were also found in the current data, and similar to predictors from the SPACE, MASTER and RADAR studies. [12–14] LBP could not be identified as a predictor anymore. In this current study the proportion of LBP ≤ 5 years was 47%, in CaFaSpA 1 only 38%, however this difference should not bias the effect of duration. Combining the validation and development dataset has several vital advantages, i.e. creating more stable predictor effects and more accurate predictions.

For the application of the rule we propose a cut point that is related to a relative high sensitivity (75%) with a lower specificity (58%). We believe that a relative high sensitivity and thus referring many possible axSpA patients is desirable, considering axSpA is a disease where quality of life increases after the start of treatment. [34] A lower specificity comes at the cost of referring CLBP patients who do not have axSpA, creating extra work in rheumatology practices. However taking into account the impact of axSpA on work participation [3], referring a relative small amount of false positive CLBP patients might even be cost-effective.

A point of discussion is that we used the ASAS criteria to define our outcome definition, namely axSpA. Classification and diagnostic criteria serve a different purpose. The difficulty in the field of axSpA is that there are no diagnostic criteria, there are only classification criteria. We believe that classification and diagnostic criteria have a substantial overlap, and that a diagnosis is almost equal to making a classification in an individual patient. [35] Moreover, classification criteria are more stringent than diagnostic criteria which is also illustrated by two cohorts who compared the diagnosis of a rheumatologist to the ASAS criteria. In the SPACE study were 65 patients diagnosed with axSpA or AS by a rheumatologist. Of these 65 patients were only 55 also classified by the ASAS criteria. [12] In the DECLIC study were 425 patients diagnosed as AS or axSpA, of those fulfilled 324 the ASAS criteria. [36] In both studies are the classification criteria more strict than the diagnosis by a rheumatologist. The specificity was high in both studies (SPACE study 95%, DECLIC 87%) so the fear of ‘over diagnosing’ a lot of patients by using the ASAS criteria, is proven not to be true by those two studies. We have chosen the ASAS criteria as outcome to identify patients as axSpA or no axSpA since this criteria are exactly defined and reproducible for readers, while the diagnosis by a rheumatologist is not. The main purpose of this article was to validate a referral strategy for axSpA in primary care, in this setting is a clear outcome definition desirable.

A remarkable finding in our study is the lower HLA-B27 prevalence (6.2%) in our study compared to other studies. [12, 13, 32] This makes a direct comparison between our study and others difficult. However, the HLA-B27 prevalence was comparable to our first large study in unselected CLBP patients [7] and to the study of Underwood [33], also performed in primary care CLBP patients. There is no evidence that HLA-B27 prevalence is higher among CLBP patients. Therefore we believe that the HLA-B27 prevalence in our study population marks the fact that we did not select on predefined axSpA features and that our referral rule is applicable in and generalizable to all primary care CLBP patients.

In conclusion we provide a stable and robust referral rule that may be applicable as a screening tool in primary care. The next step in the implementation of the referral rule will be, to investigate the clinical impact on GPs behaviour and patients’ outcomes.

## Supporting Information

S1 Table Description of Characteristics for Red flags of (sub)acute low back pain in 579 primary care chronic low back patients. (DOCX)
