# Supplementary material for: External Validation of a Referral Rule for Axial Spondyloarthritis in Primary Care Patients with Chronic Low Back Pain
Source: PLoS One. 2015 Jul 22;10(7):e0131963. doi: 10.1371/journal.pone.0131963 (PMC4511700; doi:10.1371/journal.pone.0131963)
Supplement: S1 Table — (DOCX) [file pone.0131963.s001.docx]

**S1. Table 1; Description of Characteristics for Red flags of (sub)acute low back pain in 579 primary care chronic low back patients**

| **Description red flag** | **Total (n=579)** | **<5 years LBP (n=270)** | **≥5 years LBP (n=309)** | **axSpA (n=95)** |
| --- | --- | --- | --- | --- |
| Age at onset back pain <20 years | 175 (30.2%) | 40 (14.8%) | 135 (43.7%) | 21 (22.1%) |
| Unexplained fever | 14 (2.4%) | 7 (2.6%) | 7 (2.3%) | 1 (1.1%) |
| Unbearable pain | 48 (8.3%) | 26 (9.6%) | 22 (7.2%) | 7 (7.4%) |
| Unexplained weight loss | 18 (3.1%) | 10 (3.7%) | 8 (2.6%) | 0 |
| Previous history of cancer | 16 (2.8%) | 6 (2.2%) | 10 (3.2%) | 2 (2.1%) |
| Feeling unwell | 122 (21.1%) | 57 (21.1%) | 65 (21.0%) | 21 (22.1%) |
| Longer than 2 months use of pain medication? | 35 (6.0%) | 16 (5.9%) | 19 (6.2%) | 8 (8.4%) |
| Constant pain | 205 (35.4%) | 94 (34.8%) | 111 (35.9%) | 35 (36.8%) |
| Not able to bend over | 80 (13.8%) | 35 (13.0%) | 45 (14.6%) | 13 (13.7%) |
| Significant motor weakness or sensory deficit | 225 (38.9%) | 102 (37.8%) | 123 (39.8%) | 40 (42.1%) |
| Loss of bladder control | 56 (9.7%) | 26 (9.6%) | 30 (9.7%) | 9 (9.5%) |
| Loss of sensation in the buttocks | 73 (12.6%) | 32 (11.9%) | 41 (13.3%) | 13 (13.7%) |
|  |  |  |  |  |
| 0 red flags | 120 (20.7%) | 74 (27.4%) | 46 (14.9) | 23 (24.2) |
| 1 red flags | 177 (30.6%) | 83 (30.7%) | 94 (30.4) | 23 (24.2) |
| ≥2 red flags | 282 (48.7%) | 113 (41.9%) | 169 (54.7%) | 49 (51.6) |
